# Editorial: Improving *Bacillus thuringiensis* Toxins for Better Pest Control

**DOI:** 10.3389/fmicb.2021.799011

**Published:** 2021-12-10

**Authors:** Tereza Cristina Luque Castellane, Manoel V. F. Lemos, Baltasar Escriche

**Affiliations:** ^1^Faculty of Agricultural and Veterinary Sciences, São Paulo State University, São Paulo, Brazil; ^2^Departamento de Genética, Instituto Universitario de Biotecnología y Biomedicina (BIOTECMED), Universitat de Valencia, Burjassot, Spain

**Keywords:** *Bacillus thuringiensis*, Cry protein, helicoverpa, spodoptera, resistance, pore-forming toxin

Although Cry toxins, produced by the bacterium *Bacillus thuringiensis* (Bt), have been used for years and advances in genetic engineering have been very well-explored, recent years have witnessed an increase in the evolution of insect resistance. In the last few years, research on resistance in insects and the interaction with other bioinsecticidal proteins has become more and more prominent within the field of Cry protein usage, since both processes contribute to increasing their activity and efficacy to control agricultural pests.

This Research Topic aims to bring new insights into the latest progress in our understanding of the mechanism of action of Cry proteins, and we focus our attention on the different new aspects of Cry protein detection and application, and at the same time, the resistance mechanisms that insects have developed to combat their action. Five original research articles and two reviews are included in this issue showing important aspects for unraveling the continuing battle between Cry toxins and insects.

A description of a profile using hidden Markov models (HMMs) turned out to be a tool to detect pesticide sequences and compare their genetic context. These procedures have been included as a promising implementation to improve Bt resources in the paper of Díaz-Valerio et al. They report this unique combination for identifying toxin genes to gain new insights into the use of biopesticides. Concerning biopesticides, Li et al. studied how plant polysaccharides modulate biofilm formation and insecticidal activities of Bt strains, thus building the basis for the construction of biofilm-active Bt biopesticides, with improved long-term field duration, which should be seen as an advantage.

The importance of Bt proteins for research on *Helicoverpa armigera* is discussed in three research articles. The article by Qi et al. focuses on resistance to Bt proteins in the cotton bollworm, *H. armigera*. These authors reported that Cry2Ab and Vip3Aa proteins are probably the most durable alternative for implantation in Bt cotton crops to control populations of *H. armigera* that show some resistance to Cry1Ac. They observed that significant positive cross-resistance to Cry1Fa occurred in three of the four strains tested, but not to Cry2Ab or Vip3Aa produced by whichever strain. Thus, Cry2Ab and Vip3Aa are likely to be of value in increasing both the efficacy and durability of Bt cotton against populations of *H. armigera* that have some resistance to Cry1Ac.

A second study was reported by Wei et al. which evidenced that suppression of calcineurin enhances the toxicity of Cry1Ac to *H. armigera*. The authors reported that a diphosphatase (an enzyme with phosphatase activity), namely, *Helicoverpa armigera* calcineurin (HaCAN) was involved in the insecticidal activity of Cry1Ac against the cotton worm, by regulating immune gene expression through the diphosphatase activity, but not by acting as a receptor, and these results provided a likely indication that HaCAN is an important candidate gene for use in the pyramid strategy by the simultaneous expression of RNAi (targeting CAN) and Cry1Ac in crops to control cotton bollworm.

The biotech exploration of insecticidal crystal proteins to manage insects as devastating as the cotton bollworm has been carried out extensively in recent years, and it was broadened in the study by Karthik et al. The major focus of the authors was toward the identification of promising transgenic cotton events which showed evidence of Bt toxin gene stable integration, inheritance, and superiority in their efficacy against *H. armigera*. These authors demonstrated that the use of the simultaneous expression of two *Bt* insecticidal crystal proteins (ICPs): Cry1AcF, a hybrid toxin produced by domain swapping, and Cry2Aa (produced by codon modification) for the management of *H. armigera* in transgenic cotton is an efficient form to overcome the issue of insect resistance. Therefore, this study was another way to confirm and determine that the amalgamation of several tools and strategies is necessary for the effective control of *H. armigera*. In previous reports from this group of researchers, ~2% of promising cotton transformants could be identified using the methodology. The improved performance of the general population of T2 and T3 generation plants to resist *H. armigera* supported the rigorous molecular and bio-efficacy explored in the paper published on this topic. In the future, the set of promising events identified in the study should be used in cotton improvement programs for the management of wild insects.

Based on the reports about the evolution of resistance, Liu et al. reviewed the latest work on the insecticidal mechanisms of *Bacillus thuringiensis* Cry toxins and the mechanisms of insect resistance against Bt entomopathogenic proteins. In addition to Cry proteins, many researchers have already demonstrated that Bt strains secrete Vip proteins (vegetative insecticidal protein) during their vegetative growth phase, and these proteins do not share any structural and sequence homology with Cry proteins. Thereby, they are considered a remarkable complement or supplement source of toxins to Cry proteins in crop resistance management and protection. Currently, several commercial Bt crops combine Vip3 and Cry proteins.

The topic is completed considering the review by Gupta et al. on recent advances in research on Vip proteins, to improve the Bt application as a general biocontrol agent.

The papers published in the present Special Issue: *Improving Bacillus thuringiensis Toxins for Better Pest Control* can be considered a frontier contribution to improve the Bt application to obtain better and lasting efficacy.

## Author Contributions

TC wrote the first draft of the editorial. TC, ML, and BE contributed to added some other parts and review this Editorial of the Research Topic on Frontiers that they edited in 2021. All the topic editors agree on this final version. All authors contributed to the article and approved the submitted version.

## Funding

This work was supported by the Spanish Ministry of Science and Innovation (Ref. RTI2018-095204-B-C21 and PTA2017–14403-I), co-funded by EU FEDER funds and by a grant from the Generalitat Valenciana (PROMETEO/2020/010). This work was carried out with the support of the Conselho Nacional de Desenvolvimento Científico e Tecnológico (Ref. 435574/2018-3).

## Conflict of Interest

The authors declare that the research was conducted in the absence of any commercial or financial relationships that could be construed as a potential conflict of interest.

## Publisher's Note

All claims expressed in this article are solely those of the authors and do not necessarily represent those of their affiliated organizations, or those of the publisher, the editors and the reviewers. Any product that may be evaluated in this article, or claim that may be made by its manufacturer, is not guaranteed or endorsed by the publisher.

